# 

**Published:** 1891-06-13

**Authors:** 


PRICE ONE PENNY.
No. 246, Vol. X.—June 13th, 1891.
[Registered at the General Post Office as a Newspaper for transmission in the United Kingdom and Abroad.]

WITH EXTRA NURSING SUPPLEMENT.
Per Annum, 6s. 6d.; post free.
Monthly Parts, 6d.; post free 8d.
Ditto per Annum, 8s., post free.

### THE HOSPITAL

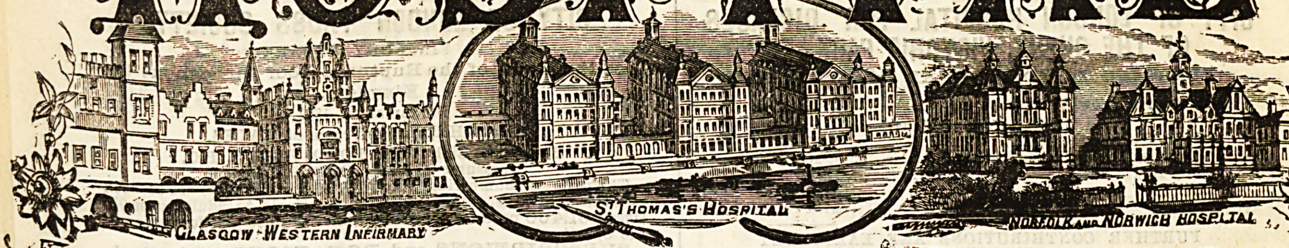

A WEEKLY JOURNAL OF

### Science, Medicine, Nursing and Philanthropy.

### SATURDAY, JUNE 13th, 1891.

**The Disestablishment of the Poor House** - 121
**Passing Topics.**—The Ex-Premier and the Poet - 122
**The Doctor's Armchair.**—Fathers Ancient and Modern - 123
**Everybody's Page** - 124
**The History and Capabilities of Herbal Simples.** By W. T. Fernie, M.D.—XXXII.—Strawberries - 125
**Notes and News** - 126
**General Charities** - 127
**Scraps and Gleanings** - 127
**Annotations.**—Sixteen Hours a Day—A Home for the Dying—Medical Fashions - 128
**The Practitioners' Mirror and Retrospect**—
Medicine.—Syringomyelia.—I. - 129
Surgery.—Treatment of Appendicitis - 129
Therapeutics.—The Aniline Dyes - 130
New Drugs, Appliances, and Things Medical.—A New Bedlift. 131; Goodman's Patent Surgical Bandages Pin—Wodderspoon and Co.'s Temperature and Diet Charts - 132
**The Indian Medical Service** - 132
**The Student of Medicine**—Editorial Notes—Presentations—Appointments—Scholarships and Prizes—Pass Lists

### EXTRA SUPPLEMENT—THE NURSING MIRROR.

**En Passant.**—Asylum Attendants—Steyning Again—The Royal Red Cross—Cumberland Infirmary - lix

**A Year in a German Hospital.**—Part II. - lx

**For Reading to the Sick**—Immortality - lxi

**Hospital Nurses and Their Work.**—From a Patient's Point of View - lxii

**Short Items** - lxii

**Examination Questions—Notes and Queries** - lxiii

**Everybody's Opinion.**—Servants and Mistresses—A Holiday Home - lxiii

**The Plain Path—Wants and Workers** - lxiii

**Off Duty.**—Modern Superstitions - lxiv

**To Nurse Angela** - lxiv

**The Nurses' Bookshelf.**—Notes on Surgery for Nurses—Another Dictionary—A Concise Pamphlet - lxiv

**Amusements and Relaxation** - lxiv

